# Waterpipe and cigarette tobacco smoking among Palestinian university students: a cross-sectional study

**DOI:** 10.1186/s12889-017-4524-0

**Published:** 2017-07-10

**Authors:** Marina Tucktuck, Rula Ghandour, Niveen M. E. Abu-Rmeileh

**Affiliations:** Institute of Community and Public Health, Birzeit University, Birzeit, occupied Palestinian territory

**Keywords:** Waterpipe tobacco smoking, Cigarette smoking, University students, Gender, Public health, Youth, Occupied Palestinian territory

## Abstract

**Background:**

During the last two decades, waterpipe tobacco smoking (WTS), also known as hookah, witnessed a global increase in use, especially among youth. Little information is known about the burden of WTS among Palestinian youth. A cross-sectional study was conducted to estimate the prevalence of WTS and cigarette smoking and explore the associated factors among a sample of Palestinian university students.

**Methods:**

1891 students, from five Palestinian universities in the West Bank and Gaza Strip, completed a self-administered, web-based survey in 2014–2015. The questionnaire, which was based on the Global Adults Tobacco Survey (GATS), had questions on WTS and cigarette smoking patterns and socio-demographic and university-related characteristics. Binary logistic regression analyses were computed to investigate associated factors with WTS and cigarette smoking.

**Results:**

50.9% of the sample was women. The mean age was 20.1 ± 2.0. Overall, 30.0% of participants were current tobacco smokers and 33.4% reported ever smoking tobacco through a waterpipe. The prevalence of current WTS (24.4%) surpassed the prevalence of current cigarette smoking (18.0%), with a significantly higher prevalence among men compared to women. The gender gap for WTS (36.4% vs. 12.9%) was smaller than that for cigarette smoking (32.8% vs. 3.6%). Binary logistic regression models for the total sample (men and women) revealed that men were more likely to be current waterpipe and cigarette tobacco smokers compared to women (AOR = 4.20, 95% CI = 3.22–5.48, and AOR = 10.91, 95% CI = 7.25–16.42, respectively). Geographic area of residence, faculty of study and self-reported academic achievement were also associated with the likelihood of being current waterpipe and cigarette tobacco smokers.

**Conclusion:**

A high prevalence of WTS was reported among our study sample, and it surpassed the prevalence of cigarette smoking. Interventions to curb the practice of tobacco smoking among Palestinian youth should be tailored differently to WTS and cigarette smoking, be gender-sensitive and specific and target the regional variation in the smoking behavior.

**Electronic supplementary material:**

The online version of this article (doi:10.1186/s12889-017-4524-0) contains supplementary material, which is available to authorized users.

## Background

Tobacco smoking is one of the leading causes of preventable death worldwide and a modifiable risk factor for the global rise in non-communicable diseases [1–5]. The World Health Organization (WHO) estimates that by the year 2030, more than 8 million deaths will be attributable to tobacco smoking, with the added burden of tobacco-specific morbidities targeting the cardiovascular and pulmonary systems [1, 6]. In the last two decades, the global epidemiological gradient of tobacco smoking began to change with new and alarming trends in waterpipe tobacco smoking (WTS) [7–9]. WTS, or hookah, as it is commonly known in some parts of the world, is a method of smoking that involves the inhalation of tobacco smoke once it passes through water [8]. While WTS is a centuries-old tradition that has been practiced for approximately 400 years, nowadays, WTS is gaining worldwide popularity among adolescents and university students [7, 10, 11]. It is believed that the increased attention geared towards WTS, particularly by youth, is the result of the introduction of flavored muassel in the 1990s, the WTS social acceptability and cultural ties, the role of the internet and social media and the lack of WTS-specific regulations [8, 9, 12]. The ubiquitous practice of WTS among adolescents and young adults prompt worrisome future health outcomes and could prelude to an emerging new strain in the global tobacco epidemic [7].

Recent epidemiological data are capturing an upward trend in the prevalence of WTS in various settings. Data from the Global Youth Tobacco Survey among adolescents (13–15 years old) reported that the highest past 30-days prevalence of WTS was in Lebanon (36.9%), followed by the West Bank in the occupied Palestinian territory (oPt) (32.7%) and Latvia (22.7%) [13]. The National Youth Tobacco Survey among U.S. students (11–18 years old) also reported an increase in the prevalence of WTS from 4.1% to 9.4% over the period 2011–2014 [14]. Among university students, global and regional data reflect a high prevalence of WTS and a higher prevalence of WTS compared to cigarette smoking. For instance, the prevalence of current, regular or past 30-days use of WTS reached as high as 20.0% in the U.S. (2010–11), 14.4% in the U.K. (2013–14) and 32.7% in Turkey (2008–09). In addition, current WTS prevalence among university students reached 23.5% (vs. 10.9% for cigarette smoking) in Syria (2006–07), 29.5% (vs. 26.3% for cigarette smoking) in Lebanon (2009–10) and 30.0% (vs. 29.0% for cigarette smoking) in Jordan (2010) [15–19].

In the oPt, the Palestinian Ministry of Health reported that in 2014, lung cancer ranked first in mortality-leading cancers [20], in part due to tobacco smoking [21]. Data on tobacco smoking from the Palestinian Central Bureau of Statistics (PCBS) revealed that in 2010, 22.5% and 15.4% of adults (≥18 years old) and youth (15–29 years old), respectively, were current tobacco smokers [22]. Among Palestinian youth (15–29 years old), the 2015 Youth Survey revealed that 23.5% of youth smoked tobacco (men, 40.9% vs. women, 5.4%) [23]. While preliminary data on the prevalence of tobacco smoking in the oPt are available, there are important gaps in the current literature that hinder our ability to fully understand the WTS phenomenon and formulate appropriate tobacco interventions. For instance, available PCBS data lack clear time-trend changes in the prevalence of WTS. In addition, most studies in the oPt focus on cigarette smoking or overall tobacco smoking, without specifying the prevalence of WTS. Furthermore, studies on tobacco smoking have only targeted either the West Bank or the Gaza Strip. Lastly, available studies on WTS behavior among university students are limited in the scope of faculties of study and year at university of students as well as number of universities under study.

Given the current gaps in the literature and the WHO’s recommendation to strengthen tobacco smoking surveillance and monitoring among various groups [3, 5], there is a necessity to understand the epidemiology of WTS in the oPt and investigate whether it is a public health concern among university students. Therefore, this study aimed to estimate the current prevalence of WTS among a sample of university students in the West Bank and Gaza Strip and compare it with the current prevalence of cigarette smoking. In addition, the study aimed to investigate possible associated factors with current WTS and cigarette smoking. It is hoped that this research will act as a baseline study for the national prevalence of WTS among Palestinian university students and inform WTS-specific interventions in the oPt. It is also hoped that the results of this research can place the prevalence of WTS within the regional and global context, so that future comparisons can be made.

## Methods

### Study design, setting and sample

A cross-sectional exploratory study was conducted among students in five Palestinian universities in the West Bank and Gaza Strip during the 2014–2015 academic year. The selection criterion for the universities was based on region (West Bank and Gaza Strip), geographic area of residence (north, central and south of the West Bank and central Gaza Strip) and size of the student body. The selection criterion also took into consideration the widest possible range of faculties the universities operate, including arts and humanities, sciences and health sciences. The Arab American University of Jenin (AAUJ) (north), Birzeit University (central), and Hebron University (south) in the West Bank, and Al-Azhar University and the Islamic University-Gaza, both of which are in Gaza city, agreed to participate in the study (*N* = 55,959) [24].

The sampling frame comprised of all enrolled, full-time, undergraduate students at the selected universities. Sample size (SS) calculation was estimated at the university level. Based on the total student population at the participating universities, the infinite SS equation (SS = (z^2^ * p * q) / d^2^) was used, with a confidence interval of 95% (α = 0.05), hence a z-score of 1.96, a predicted WTS prevalence of *p* = 0.5 and an absolute precision of d = 0.05. A SS of *n* = 384 student was required from each university. To account for a non-response of 25%, a SS of 480 was requested from the participating universities. Equal SS was applied to ensure sufficient numbers from each university and to meet the research objective of exploring the WTS and cigarette smoking behavior among university students. The final SS was 1891 students with an overall survey completion rate of 79.2%. One university did not meet the required SS within the allotted time-frame for data collection.

### Data collection

Data were mainly collected using the Arabic-translated and standardized Global Adults Tobacco Survey (GATS) [25]. The GATS was selected for our study as the WHO urges countries to use this standard tool to maintain consistency and comparability in the monitoring and surveillance of tobacco use [25]. Since our study targeted university students, GATS was a suitable choice as it is designed to be administered for all men and women, 15 years of age or older [25]. The final questionnaire was piloted in two ways, web-based and paper-based, at a Palestinian university not included in the study. The questionnaire consisted of eight sections that measure socio-demographic and university-related characteristics, WTS and cigarette smoking patterns, smoking environment, self-rated health status, knowledge, perceptions, attitudes and opinions on WTS as well as use of electronic-cigarettes. After receiving ethical approval for conducting the study from the Research Ethics Committee of the Faculty of Graduate Studies at Birzeit University, the fieldwork was carried out between February–April of 2015. Participants were invited to fill out a self-administered, web-based survey. The survey link was placed on the student-university electronic portal page (in compliance with the universities’ policies) to ensure participation of students from the selected universities only. An overview of the study and its objectives were presented to the students prior to obtaining their online consent (by answering ‘yes’ to the question: *do you agree to participate in this study?*). Participants were ensured complete confidentiality on their personal information and anonymity of their responses. It was explained to them that their participation was voluntary, that they can withhold from answering any question, and that they could choose to withdraw from the study at any point. An intensive follow-up was carried out in conducting the study. A qualitative focus-group discussion (*n* = 7) and in-depth interviews (*n* = 10) were conducted with students at Birzeit University in July 2015 and October 2015, respectively, to gain a better understanding of the results of the study.

### Outcome measures and statistical analysis

In the GATS, current tobacco smokers correspond to the percentage of respondents who currently smoke tobacco [25]. In this study, the primary outcome variable was whether the respondent was a current waterpipe tobacco smoker and the secondary outcome variable was whether the respondent was a current cigarette smoker. The primary outcome question was: *‘Do you currently smoke waterpipe on a daily, less than daily, or not at all?’* Response options included: *daily, less than daily and not at all*. According to the GATS, daily means smoking at least one tobacco product every day or nearly every day over a period of a month or more [25]. In the analysis, current WTS status was recoded into a current (daily or less than daily) waterpipe tobacco smoker and not a current waterpipe tobacco smoker. The same definition was applied to the secondary outcome variable. The standardized prevalence (standardized to university population size) was reported for each university. The covariates included a number of socio-demographic and university-related characteristics.

The prevalence of current WTS (primary outcome variable) and current cigarette smoking (secondary outcome variable) was reported as a proportion (%) of the total sample. For descriptive categorical variables, proportions were computed and for descriptive continuous variables, data were presented in terms of means and standard deviation (SD) or medians and interquartile range (IQR). Chi-square analysis (χ^**2**^) was used to test for statistical significance between our outcomes variables and selected covariates. The stepwise regression method was used for the logistic regression analysis, which was computed to estimate the adjusted odds ratio (AOR) for selected covariates with our outcomes variables. Study findings were presented based on our objective to compare WTS and cigarette smoking among the study sample. Given the results of the chi-square analysis on the variation of current WTS and current cigarette smoking with gender, separate regression models were computed for men and women. Three binary logistic regression models, for total sample, men sample only and women sample only, were computed for our primary and secondary outcome variables (for a total of 6 regression models). The regression models were adjusted for gender and age and included the following covariates: locality type (urban, rural, camp), geographic area of residence, employment status, living arrangement (with family or other), parental highest educational attainment, self-rated financial status, faculty of study and self-reported academic achievement (measured by grade point average, GPA). The AOR and the 95% confidence interval (CI) were reported for the factors associated with our outcome variables. Statistical significance was defined at *p* < 0.05. Data were analyzed using the Statistical Package for the Social Sciences (SPSS) version 22.0.

## Results

### Sample characteristics

Responses of 1891 students were analyzed. The sample was 50.9% women and the mean age was 20.1 years old (SD = 2.0). Among the study sample, more than half (59.3%) were West Bank residents and 40.7% were from the Gaza Strip. At the time of the survey, more than two thirds of the sample (87.9%) lived with their families, 91.4% were single and 13.9% were employed. As for university-related characteristics, more than half (54.3%) were students in the faculties of arts and humanities, followed by 28.1% in the faculties of sciences and 17.6% in the faculties of health sciences. Other socio-demographic and university-related characteristics of the study sample, stratified by gender, are presented in Table 1.

**Table 1:** Baseline socio-demographic and university-related sample characteristics, stratified by gender (*n* = 1891)

Characteristic	Total (*n* = 1891)		Women (*n* = 962)	Men (*n* = 929)
	N	%	%	%
Locality				
Urban	1108	58.6	61.7*	55.3*
Rural	590	31.2	29.3	33.2
Camp	193	10.2	8.9	11.5
Geographic area of residence				
North West Bank	370	19.6	13.4***	25.9***
Central West Bank	360	19.0	20.5	17.5
South West Bank	391	20.7	29.7	11.3
North Gaza Strip	121	6.4	4.6	8.3
Central Gaza Strip	514	27.2	24.6	29.8
South Gaza Strip	135	7.1	7.2	7.1
Living arrangement				
With family	1660	87.9	90.7***	84.9***
Other	229	12.1	9.3	15.1
Employment status				
Employed	262	13.9	5.3***	22.7***
Not employed	1629	86.1	94.7	77.3
Father’s highest educational attainment				
Less than high school	429	22.7	23.4	22.0
High school	439	23.2	24.1	22.3
Higher than high school	1023	54.1	52.5	55.8
Mother’s highest educational attainment				
Less than high school	580	30.7	29.5	31.9
High school	604	31.9	32.3	31.5
Higher than high school	707	37.4	38.1	36.6
Self-rated financial status				
Poor	556	30.5	26.2***	34.9***
Good	686	37.6	39.0	36.2
Very good	582	31.9	34.8	28.9
University attended				
AAUJ ^a^	384	20.3	13.9***	26.9***
Birzeit University	384	20.3	21.0	19.6
Hebron University	355	18.8	28.9	8.3
Al-Azhar University	384	20.3	15.4	25.4
Islamic University	384	20.3	20.8	19.8
Current year at university				
First	550	29.3	31.2*	27.4*
Second	434	23.1	22.8	23.5
Third	433	23.1	24.3	21.8
Fourth and above	460	24.5	21.8	27.4
Current faculty of study				
Arts & humanities	1005	54.3	56.7***	51.9***
Sciences	520	28.1	22.4	34.0
Health sciences	325	17.6	21.0	14.1
Self-reported academic achievement				
GPA^b^ ≤ 69.9	185	10.0	6.9***	13.2***
GPA 70.0–79.9	966	52.0	48.4	55.7
GPA ≥ 80.0	708	38.1	44.8	31.1

^a^ *AAUJ* Arab American University of Jenin

^b^ *GPA* Grade point average

*Significant at the <0.05 level

***Significant at the <0.001 level

### Prevalence and patterns of WTS and cigarette smoking

**Fig. 1:**
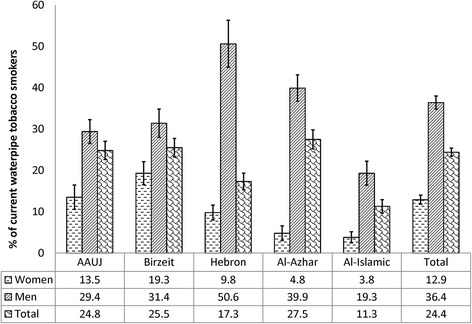
The standardized prevalence of current waterpipe tobacco smoking, stratified by gender, by university attended (*n* = 1891)

**Fig. 2:**
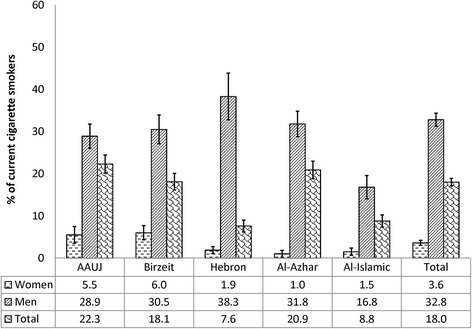
The standardized prevalence of current cigarette smoking, stratified by gender, by university attended (*n* = 1891)

**Fig. 3:**
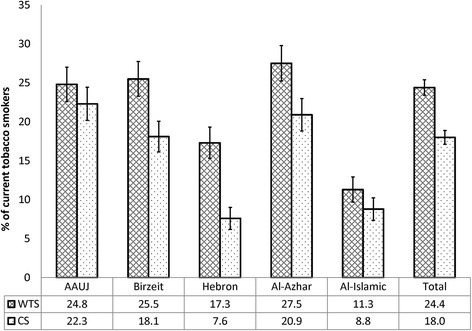
The standardized prevalence of current waterpipe tobacco smoking (WTS) and cigarette smoking (CS), by university attended (*n* = 1891)

Overall, 30.0% of the study sample were current (daily or less than daily) tobacco smokers, attributed to WTS and cigarette smoking. Of the total sample, 12.4% were dual waterpipe/cigarette smokers, 12.0% were exclusive waterpipe tobacco smokers and 5.6% were exclusive cigarette smokers. The prevalence of current WTS among the study participants was 24.4% and it predominated among men (36.4%) in comparison to women (12.9%), (χ^**2**^ = 141.3 and *p*-value <0.001) (Fig. 1). Among the study sample, 33.4% of those who were not current waterpipe tobacco smokers had ever tried WTS. As for the prevalence of current cigarette smoking of 18.0%, it was considerably higher among men (32.8%) in comparison to women (3.6%), (χ^**2**^ = 273.1 and *p*-value <0.001) (Fig. 2). Noteworthy was the smaller gender gap for WTS compared to the gender gap for cigarette smoking. The highest prevalence of WTS was observed among students attending Al-Azhar University in Gaza city, while the highest prevalence of cigarette smoking was among students attending the AAUJ in the northern geographic area of the West Bank (Fig. 3). Overall, WTS seemed to mostly be a ‘less than daily’ practice as opposed to ‘daily’ (20.3% vs. 4.1%) and cigarette smoking was mostly a ‘daily’ practice as opposed to ‘less than daily’ (12.6% vs. 5.3%). The median age of initiation among current waterpipe tobacco smokers was 17.0 years (IQR = 16–18), compared to a lower median age of initiation among current cigarette smokers of 16.0 years (IQR = 15 – 18). On average, participants reported that the mean duration of their last WTS session was 63.0 min (SD = 46.2).

### Factors associated with WTS and cigarette smoking

Table 2 presents the statistically significant associations between current WTS and individual characteristics (as well as between current cigarette smoking and individual characteristics) for the total sample, men sample only and women sample only. For the total sample (men and women), current WTS was significantly associated with gender, age (unadjusted OR = 1.1, 95% CI = 1.0–1.1), locality type, geographic area of residence, living arrangement, employment status, self-rated financial status and self-reported academic achievement. As for current cigarette smoking for the total sample, it was significantly associated with gender, age (unadjusted OR = 1.1, 95% CI = 1.1–1.2), geographic area of residence, living arrangement, employment status and self-reported academic achievement.

**Table 2:** Socio-demographic and university-related associations with current waterpipe tobacco smoking and cigarette smoking, stratified by gender (*n* = 1891)

Characteristic	Total (*n* = 1891)		Women (*n* = 962)		Men (*n* = 929)	
	WTS ^§^	CS ^£^	WTS	CS	WTS	CS
	n (row %)	n (row %)	n (row %)	n (row %)	n (row %)	n (row %)
Locality						
Urban & Camp	428 (25.2)*	305 (18.0)	120 (13.7)*	34 (3.9) ^**C**^	308 (37.5)	271 (33.0)
Rural	34 (17.6)	35 (18.1)	4 (4.7)	1 (1.2)	30 (28.0)	34 (31.8)
Geographic area of residence						
North West Bank	139 (37.6)***	125 (33.8)***	21 (16.3)***	8 (6.2)** ^**C**^	118 (49.0)***	117 (48.5)***
Central West Bank	107 (29.7)	73 (20.3)	44 (22.3)	14 (7.1)	63 (38.7)	59 (36.2)
South West Bank	99 (25.3)	52 (13.3)	49 (17.1)	10 (3.5)	50 (47.6)	42 (40.0)
Gaza Strip	117 (15.2)	90 (11.7)	10 (2.9)	3 (0.9)	107 (25.5)	87 (20.7)
Living arrangement						
With family	381 (23.0)***	264 (15.9)***	109 (12.5)	25 (2.9)*** ^**C**^	272 (34.5)**	239 (30.3)***
Other	81 (35.4)	76 (33.2)	15 (16.9)	10 (11.2)	66 (47.1)	66 (47.1)
Employment status						
Employed	99 (37.8)***	99 (37.8)***	6 (11.8)	4 (7.8) ^**C**^	93 (44.1)**	95 (45.0)***
Not employed	363 (22.3)	241 (14.8)	118 (13.0)	31 (3.4)	245 (34.1)	210 (29.2)
Father’s highest educational attainment						
High school & above	368 (25.2)	275 (18.8)	92 (12.5)	31 (4.2)	276 (38.1)*	244 (33.7)
Less than high school	94 (21.9)	65 (15.2)	32 (14.2)	4 (1.8)	62 (30.4)	61 (29.9)
Mother’s highest educational attainment						
High school & above	329 (25.1)	238 (18.2)	92 (13.6)	26 (3.8)	237 (37.4)	212 (33.5)
Less than high school	133 (22.9)	102 (17.6)	32 (11.3)	9 (3.2)	101 (34.1)	93 (31.4)
Self-rated financial status						
Poor	116 (20.9)*	103 (18.5)	23 (9.5)	7 (2.9)	93 (29.7)**	96 (30.7)
Good	168 (24.5)	122 (17.8)	50 (13.8)	10 (2.8)	118 (36.4)	112 (34.6)
Very good	166 (28.5)	101 (17.4)	49 (15.2)	17 (5.3)	117 (45.2)	84 (32.4)
Current faculty of study						
Arts & humanities	251 (25.0)	191 (19.0)	70 (13.2)	22 (4.1)	181 (38.3)	169 (35.7)*
Sciences & health sciences	204 (24.1)	140 (16.6)	52 (12.8)	12 (2.9)	152 (34.7)	128 (29.2)
Self-reported academic achievement						
GPA^**b**^ ≤ 69.9	72 (38.9)***	67 (36.2)***	15 (23.1)**	1 (1.5) ^**C**^	57 (47.5)***	66 (55.0)***
GPA 70.0–79.9	272 (28.2)	202 (20.9)	69 (15.1)	20 (4.4)	203 (40.0)	182 (35.8)
GPA ≥ 80.0	109 (15.4)	63 (8.9)	37 (8.7)	12 (2.8)	72 (25.4)	51 (18.0)

^C^One cell has expected cell count <5

^§^ *WTS* Waterpipe tobacco smoking

^£^ *CS* Cigarette smoking

^b^ *GPA* Grade point average

*Significant at the <0.05 level

**Significant at the <0.01 level

***Significant at the <0.001 level (significance was computed using chi-square analyses by comparing the proportions of smokers in each socio-demographic and university-related category)

Table 3 presents the binary logistic regression models for the total sample (men and women) for WTS and cigarette smoking. The reference category was ‘not a current waterpipe tobacco smoker,’ and ‘not a current cigarette smoker,’ thus the AOR in the model denotes the probability of being a current waterpipe tobacco smoker and a current cigarette smoker, respectively. An additional table shows the binary logistic regression models for WTS and cigarette smoking for men and women, separately [see Additional file 1].

**Table 3:** Logistic regression for current waterpipe tobacco smoking and cigarette smoking by participants’ characteristics for the total sample (*n* = 1891)

Characteristic	Waterpipe tobacco smoking	Cigarette smoking
	AOR (95% CI)	AOR (95% CI)
Age (in years) ^§^	-	1.12 (1.05–1.20)
Gender		
Women ^R^	1	1
Men	4.20 (3.22–5.48)	10.91 (7.25–16.42)
Locality		
Urban & Camp ^R^	-	1
Rural	-	1.90 (1.17–3.08)
Geographic area of residence		
Gaza Strip ^R^	1	1
North West Bank	3.20 (2.31–4.44)	3.78 (2.51–5.70)
Central West Bank	2.66 (1.91–3.72)	2.66 (1.73–4.08)
South West Bank	2.81 (1.99–3.95)	2.40 (1.51–3.83)
Living arrangement		
With family ^R^	-	1
Other	-	1.79 (1.19–2.68)
Employment status		
Not employed ^R^	-	1
Employed	-	1.54 (1.08–2.18)
Mother’s highest educational attainment		
Less than high school ^R^	-	1
High school & above	-	1.41 (1.03–1.93)
Self-rated financial status		
Poor ^R^	1	-
Good	1.33 (0.99–1.80)	-
Very good	1.68 (1.24–2.29)	-
Current faculty of study		
Sciences & health sciences ^R^	1	1
Arts & humanities	1.28 (1.00–1.63)	1.76 (1.29–2.40)
Self-reported academic achievement		
GPA^b^ ≥ 80.0 ^R^	1	1
GPA 70.0–79.9	1.79 (1.36–2.36)	2.15 (1.51–3.08)
GPA ≤ 69.9	2.94 (1.96–4.41)	4.50 (2.78–7.30)

^R^Reference category

*AOR (95% CI)* Adjusted odds ratio (95% confidence interval)

^§^Continuous variable, AOR reflects the change in age (1 year) following one unit change in our outcome variables

^b^ *GPA* Grade point average

For the total sample (men and women), some noteworthy results of the binary logistic regression for current WTS revealed that men were more likely to be current waterpipe tobacco smokers compared to women (AOR = 4.2, 95% CI = 3.2–5.5). Students who had a very good self-rated financial status were more likely to be current waterpipe tobacco smokers in comparison to those who had a poor self-rated financial status (AOR = 1.7, 95% CI = 1.2–2.3). As for current cigarette smoking for the total sample, the regression model revealed that men were also more likely to be current cigarette smokers compared to women (AOR = 10.9, 95% CI = 7.3–16.4) and cigarette smoking tended to increase by increasing age (unadjusted OR = 1.1, 95% CI = 1.1–1.2). In addition, students who lived in rural settings were more likely to be current cigarette smokers compared to those who resided in urban/camp settings (AOR = 1.9, 95% CI = 1.2–3.1); those who did not live with their families were more likely to be current cigarette smokers compared to those who lived with their families (AOR = 1.8, 95% CI = 1.2–2.7); students who were employed at the time of the survey were more likely to be current cigarette smokers in comparison to those who were not employed (AOR = 1.5, 95% CI = 1.1–2.2); and students whose mother had attained high school degree and above were more likely to be current cigarette smokers compared to those whose mother had attained less than a high school degree (AOR = 1.4, 95% CI = 1.0–1.9).

For the men sample only, the regression model for current WTS revealed that students who were from the northern, central and southern geographic areas of the West Bank were more likely to be current waterpipe tobacco smokers in comparison to those from the Gaza Strip (AOR = 2.6, 95% CI = 1.8–3.7, AOR = 1.5, 95% CI = 1.0–2.3 and AOR = 2.3, 95% CI = 1.5–3.7, respectively). Students who had a very good or good self-rated financial status were more likely to be current waterpipe tobacco smokers in comparison to those who had a poor self-rated financial status (AOR = 1.8, 95% CI = 1.3–2.7 and AOR = 1.3, 95% CI = 0.9–1.9, respectively). Students who had a self-reported academic achievement (GPA) of ≤69.9 and 70.0–79.9 were more likely to be current waterpipe tobacco smokers in comparison to students who had a self-reported academic achievement (GPA) of ≥80.0 (AOR = 2.9, 95% CI = 1.8–4.7 and AOR = 1.9, 95% CI = 1.4–2.7, respectively). As for the regression model for current cigarette smoking among men, it was found that students who were from the northern, central and southern geographic areas of the West Bank were also more likely to be current cigarette smokers compared to those from the Gaza Strip (AOR = 4.5, 95% CI = 3.0–6.8, AOR = 2.4, 95% CI = 1.5–3.9 and AOR = 3.0, 95% CI = 1.8–5.1, respectively) and those who lived in rural settings were more likely to be current cigarette smokers compared to those who resided in urban/camp settings (AOR = 2.1, 95% CI = 1.2–3.5). Students who were studying in the faculties of arts and humanities were more likely to be current cigarette smokers compared to those at the faculties of sciences and health sciences (AOR = 1.4, 95% CI = 1.0–2.0) and those who had a self-reported academic achievement (GPA) of ≤69.9 and 70.0–79.9 were more likely to be current cigarette smokers compared to those whose self-reported academic achievement (GPA) was ≥80.0 (AOR = 5.9, 95% CI = 3.5–10.0 and AOR = 2.4, 95% CI = 1.6–3.5, respectively). Cigarette smoking also tended to increase by increasing age (unadjusted OR = 1.2, 95% CI = 1.1–1.2) for men.

As for the women sample only, the regression model for current WTS revealed that students who were from the northern, central and southern geographic areas of the West Bank were more likely to be current waterpipe tobacco smokers compared to those from the Gaza Strip (AOR = 7.2, 95% CI = 3.2–16.5, AOR = 9.5, 95% CI = 4.6–19.7 and AOR = 6.4, 95% CI = 3.2–13.0, respectively) and students who had a self-reported academic achievement (GPA) of ≤69.9 and 70.0–79.9 were more likely to be current waterpipe tobacco smokers compared to students who had a self-reported academic achievement (GPA) of ≥80.0 (AOR = 3.7, 95% CI = 1.8–7.6 and AOR = 1.8, 95% CI = 1.1–2.8, respectively). For current cigarette smoking, the regression model for women found that students who were from the northern, central and southern geographic areas of the West Bank were also more likely to be current cigarette smokers compared to those from the Gaza Strip (AOR = 5.6, 95% CI = 1.3–24.1, AOR = 8.7, 95% CI = 2.5–30.8 and AOR = 4.4, 95% CI = 1.2–16.2, respectively) and students whose father’s highest educational attainment was high school degree and above were more likely to be current cigarette smokers compared to those whose father’s highest educational attainment was less than a high school degree (AOR = 4.8, 95% CI = 1.1–20.3).

## Discussion

### Main findings

WTS seems to be a more popular method of tobacco smoking compared to cigarette smoking among Palestinian university students in our study sample (24.4% vs. 18.0%). In comparison to other studies in the oPt, our reported WTS prevalence was considerably higher than the prevalence reported by PCBS among enrolled university students (17–25 years old), which increased from 0.5% in 2000 to 2.0% in 2010. This finding could be due to the fact that PCBS estimates are based on proxy self-reported smoking. As for the reported prevalence of ‘other tobacco products,’ mainly attributed to WTS, among Palestinian health students, our current WTS prevalence fell within the documented range of 12.3% to 30.9% (2007) [26].

In relation to documented WTS prevalence among university students elsewhere, our results were overall consistent with other studies. For instance, our WTS prevalence surpassed the reported current WTS prevalence of 5.6% in the United Arab Emirates (2005) [27]. Additionally, it was lower than the current WTS prevalence of 36.3% reported among university students in Saudi Arabia (2010) [28], the 40.0% among university students in South Africa (2013) and the 37.8% among women university students in Egypt (2007) [29, 30]. In comparison to the prevalence of WTS in Western countries, a study among U.S. university students had a lower reported WTS prevalence of 12.5% compared to our reported prevalence (2011) [31]. Another study among medical students in the U.K. reported a WTS prevalence of 11.0% and a 6.3% cigarette smoking prevalence [32], both of which were lower than our reported prevalence for WTS and cigarette smoking, respectively. This evidence reveals varying degrees of WTS use and popularity in different contexts, which could partially be due to existing tobacco laws and in part due to the social and cultural acceptability of WTS among youth [33, 34]. In addition, when considering the higher reported prevalence of WTS among our study sample in comparison with the prevalence in countries such as the U.S. and the U.K., it is worth pointing out that WTS has been linked to social class and prestige in the Middle Eastern culture, while the opposite was true in Western countries [8, 9, 19, 35–38]. Some studies in the U.S. have also found a higher WTS prevalence among university students from Arab or Middle Eastern decent in comparison to students from other backgrounds [31, 39]. These studies grant support to both the cultural and symbol status associated with WTS among Middle Eastern students. Regular monitoring of WTS among young people, especially from Middle Eastern backgrounds, needs to be in place to curb the WTS prevalence from escalating.

Among our study sample, the higher prevalence of WTS compared to cigarette smoking supports the increased popularity of WTS among youth, as an acceptable alternative to cigarette smoking. This is also supported by the tolerability of WTS among families in the Arab culture [8, 9]. The higher prevalence of WTS could also reflect an emerging WTS epidemic among youth in the Eastern Mediterranean Region. In the oPt, results from the 2015 youth survey revealed that 50.0% of the sample (15–29 years old) believes that the major health issues they face are induced by behaviors such as tobacco smoking [23]. Still, many youth perceive WTS as less harmful than cigarette smoking and show a lack of intent to quit [40–42]. These findings could elicit an alarm towards an increased risk of continuation of WTS into adulthood, thus contributing to the WTS epidemic.

The smoking profile and patterns of our study sample were overall consistent with the published literature [8, 9, 43]. For instance, age of initiation of WTS was slightly higher than for cigarette smoking. The age of initiation coincides with a transitional period from high school to university, a period thought to involve many behavioral changes, including tobacco smoking [44–46]. Our study design did not allow us to study this transitional period, thus future research should target youth during this phase. The average WTS duration among our study sample of 63 min fell within the range reported in other studies [8, 9, 33, 47]. The long duration of a WTS session can be due to the cultural aspect of WTS where it is viewed as a pleasurable social activity that brings together family and friends. In some studies, participants expressed that the waterpipe availability in restaurants and cafes at an affordable cost further encourages them to smoke waterpipe. In addition, others brought attention to the role of sensory qualities of WTS and innovative designs of the waterpipe instrument in encouraging continued practice of WTS. These testimonies could support the longer duration of a WTS session in comparison to cigarette smoking, which is viewed as an individualistic and habitual practice with no ties to any social or cultural aspect [8, 9, 33, 37, 47, 48].

Consistent with other studies [9, 17, 49, 50], the gender-gradient of WTS and cigarette smoking was evident among our study sample, with a higher smoking prevalence among men in comparison to women. This could be explained by the socio-cultural beliefs about tobacco smoking, in which there still exists a distinction between men and women smoking habits; this finding was corroborated during our interviews. However, our results also reflect a smaller gender gap in WTS compared to cigarette smoking; some studies have indeed alluded that the gender gap for WTS is absent or diminishing [9, 17, 49, 50]. Among our study sample, while men were about 11 times more likely to be current cigarette smokers compared to women, for WTS, men were about 4 times more likely to be current waterpipe tobacco smokers compared to women. This finding could be due to the cultural perception that WTS is more tolerated for women compared to cigarette smoking [8, 9]. Qualitative studies have addressed the various social attitudes surrounding women’s WTS such as, “expression of eagerness for more liberal choices,” which could also explain the changing gender gap in WTS [47]. These findings call for tailored WTS-interventions for men and women and a re-assessment of the social attitudes surrounding men and women smoking behaviors.

Our finding that students who live in the West Bank had higher odds of being current waterpipe and cigarette tobacco smokers compared to those from the Gaza Strip was consistent with some studies that observed regional variation in the prevalence of tobacco smoking [10, 51–53]. Among our study sample, the cultural aspect of WTS, which is characterized by social, cultural and familial acceptability or, lack thereof, could explain the regional variation in the practice of WTS. In addition, the presence and differential access to WTS cafes around universities in different areas of the oPt could also explain the observed regional variation in the prevalence of WTS. In our study sample, 57.1% indicated that during their last WTS session, they had smoked waterpipe at a restaurant or coffee-shop. This finding was also corroborated during our interviews, highlighting the presence of single-gendered and student-friendly cafes in different geographic areas of the oPt. When considering the results in the Gaza Strip, caution should be taken into account when comparing the prevalence of WTS between the West Bank and Gaza Strip. Many studies, including two in the oPt, have linked exposure to conflict and violence to an increased risk of tobacco smoking [54–57]. However, due to a dearth of documentation on the recent impact of the Gaza war on the smoking behavior of Gaza Strip residents, there is a need for further research to accurately interpret the results.

As for university-related characteristics among our study sample, students at the faculties of arts and humanities had higher odds of being waterpipe and cigarette tobacco smokers compared to those studying at the faculties of sciences and health sciences. This could be viewed in light of the type of education that students in the sciences and health sciences receive about smoking [9, 43]; further research is needed to assess the level of knowledge among students on the health risks associated with WTS. In addition, this finding supports the important role of the educational system in incorporating the effect of WTS on health in the curriculum [40], especially that a study among Palestinian youth revealed a lack of awareness on health issues, including smoking [58]. Furthermore, the finding that students with a low self-reported academic achievement had higher odds of being current waterpipe and cigarette tobacco smokers is consistent with the literature [53, 59, 60]. This could be due to the social network of friends that is created through WTS, which encourages smoking, indirectly shifting attention from studies.

### Strengths and limitations

The current study provided invaluable information on the higher prevalence of WTS compared to cigarette smoking, marking WTS as a potential public health concern among university students in the oPt. To the best of my knowledge, this is the first study on the prevalence of WTS among university students in both the West Bank and Gaza Strip and the first study to compare the prevalence and associated factors between WTS and cigarette smoking. The prevalence results can thus act as a baseline for future studies. In addition, this study is the first to utilize the core questions from the GATS, which can support future local and regional comparisons. The current exploratory study had some limitations inherent in its cross-sectional design, which is not intended for generalizations. The study has only provided a glimpse into the factors which contribute to WTS and cigarette smoking among Palestinian university students. Given our objective to explore the WTS prevalence and behavior among selected universities, it was also not our intention to select a representative sample from the university student population where the results could be generalized outside the participating universities. With the equal sample strategy, the study focused on shedding light on the current situation of WTS behavior among a sample of Palestinian university students. In addition, the use of a cross-sectional design hampers the ability to make any causal links between our outcome variables and associated factors. Lastly, participation in the study was based on self-selection, which has an inherent bias in the characteristics of the non-respondents.

## Conclusions

WTS seems to be a context, gender and region-specific phenomenon and it differs from the individualistic nature of cigarette smoking. The findings of this study have public health and policy implications that may be considered by health professionals, educators and policy-makers. In terms of national policies, the findings of this study should draw attention to the emerging WTS trend that is surpassing cigarette smoking among youth. It should prompt policy makers to adopt WTS-specific interventions to prevent the WTS trends from propagating [34]. In the oPt, existing tobacco policies are not tailored to WTS and only address cigarette smoking. The Public Health Law, issued in 2005 (No Smoking Law), dictates a ban on tobacco smoking in public places and does not allow selling of cigarettes to those who are <18 years old [61, 62]. However implementation remains weak as there is no system in place to enforce compliance or issue penalties. Thus, it is hoped that the results of the current study can provide a trigger for more concrete action plans and regulations that specifically target youth and university students in an attempt to promote healthy behaviors. In addition, it is hoped that the results would trigger continuous monitoring of the WTS behavior among Palestinian university students.

## Additional file

Additional file 1: Logistic regression for current waterpipe tobacco smoking and cigarette smoking by participants’ characteristics for men in the sample only (n = 929) and women in the sample only (n = 962). (PDF 146 kb)

## Abbreviations

AAUJ: Arab American University of Jenin
AOR: Adjusted odds ratio
CI: Confidence interval
CS: Cigarette smoking
GATS: Global Adults Tobacco Survey
GPA: Grade point average
IQR: Interquartile range
oPt: occupied Palestinian territory
OR: Odds ratio
PCBS: Palestinian Central Bureau of Statistics
SD: Standard deviation
SPSS: Statistical Package for the Social Sciences
SS: Sample size
WHO: World Health Organization
WTS: Waterpipe tobacco smoking

## References

[1] World Health Organization (2008). WHO Report on the Global Tobacco Epidemic, 2008.

[2] World Health Organization, WHO report on the global tobacco epidemic, 2011: warning about the dangers of tobacco. Geneva; 2011.

[3] World Health Organization, Global action plan for the prevention and control of noncommunicable diseases 2013–2020. Geneva; 2013.

[4] World Health Organization, WHO global report on trends in prevalence of tobacco smoking 2015. 2015.

[5] WHO Study Group on Tobacco Product Regulation (TobReg), Waterpipe tobacco smoking: health effects, research needs and recommended actions for regulators - 2nd ed. World Health Organization; 2015.

[6] El-Zaatari, ZM, Chami HA, Zaatari GS, Health effects associated with waterpipe smoking. Tob Control. 2015;24:i31-i43. 10.1136/tobaccocontrol-2014-051908 PMC4345795 25661414

[7] Maziak W (2004). Tobacco smoking using a waterpipe: a re-emerging strain in a global epidemic. Tob Control.

[8] Maziak W, et al. The global epidemiology of waterpipe smoking. Tob Control. 2015;24:i3-i12. 10.1136/tobaccocontrol-2014-051903 PMC4345835 25298368

[9] Jawad M (2013). To what extent should waterpipe tobacco smoking become a public health priority?. Addiction.

[10] Anjum Q, Ahmed F, Ashfaq T (2008). Knowledge, attitude and perception of water pipe smoking (shisha) among adolescents aged 14-19 years. J Pak Med Assoc.

[11] Knishkowy B, Amitai Y (2005). Water-pipe (narghile) smoking: an emerging health risk behavior. Pediatrics.

[12] Kheirallah KA (2016). Waterpipe tobacco smoking among Arab youth; a cross-country study. Ethnicity & disease.

[13] Jawad M, Lee JT, Millett C. Waterpipe tobacco smoking prevalence and correlates in 25 Eastern Mediterranean and Eastern European countries: cross-sectional analysis of the Global Youth Tobacco Survey. Nicotine & Tobacco Research [Abstract], 2015: p. ntv101. 10.1093/ntr/ntv101 25957438

[14] Arrazola RA (2015). Tobacco use among middle and high school students-United States, 2011-2014. MMWR. Morbidity and mortality weekly report.

[15] Jawad M (2016). Waterpipe tobacco use in the United Kingdom: a cross-sectional study among university students and stop smoking practitioners. PLoS One.

[16] Poyrazoğlu S (2010). Waterpipe (narghile) smoking among medical and non-medical university students in Turkey. Upsala J Med Sci.

[17] Almerie MQ (2008). Cigarettes & waterpipe smoking among medical students in Syria: a cross-sectional study. Int J Tuberc Lung Dis.

[18] Jradi H (2013). Cigarette and waterpipe smoking associated knowledge and behaviour among medical students in Lebanon. East Mediterr Health J.

[19] Khabour OF (2012). Waterpipe tobacco and cigarette smoking among university students in Jordan. Int J Tuberc Lung Dis.

[20] Palestinian Ministry of Health, Annual Health Report - 2014. 2014.

[21] Palestinian Central Bureau of Statistics and Palestinian Ministry of Health, The Palestinian Central Bureau of Statistics (PCBS) and the Ministry of Health (MoH) are issuing a Press Release on the occasion of International Day of Giving up Smoking (Word No Tobacco Day) on 31/5/2012. 2012.

[22] Palestinian Central Bureau of Statistics, Final Report of the Palestinian Family Survey 2010 2013: Ramallah – State of Palestine.

[23] Palestinian Central Bureau of Statistics, Youth Survey 2015 - Main Findings. 2016: Ramallah - Palestine.

[24] Ministry of Education and Higher Education, Higher Education Statistical Yearbook - 2014-2015. 2015: Ramallah - Palestine.

[25] Global Adult Tobacco Survey Collaborative Group (2011). Tobacco questions for surveys: a subset of key questions from the Global Adult Tobacco Survey (GATS).

[26] Al-Kariry M. Global Health Professions Students Survey (GHPSS) Fact Sheet 2007, West Bank and Gaza Strip. World Health Organization-Regional Office for the Eastern Mediterranean. 2010. http://www.emro.who.int/tobacco/gtss-matrix/ghpss-factsheets-reports.html#ghpss-country-reps.

[27] Mandil A (2007). Characteristics and risk factors of tobacco consumption among University of Sharjah students, 2005. East Mediterr Health J.

[28] Al Mohamed H, Amin T. Pattern and prevalence of smoking among students at King Faisal University, Al Hassa, Saudi Arabia. East Mediterr Health J. 2010;16(1):56–64. 20214159

[29] Daniels KE, Roman NV (2013). A descriptive study of the perceptions and behaviors of waterpipe use by university students in the Western Cape*,* South Africa. Tob Induc Dis.

[30] Labib N (2007). Comparison of cigarette and water pipe smoking among female university students in Egypt. Nicotine Tob Res.

[31] Abughosh S (2012). Ethnicity and waterpipe smoking among US students. Int J Tuberc Lung Dis.

[32] Jawad M (2013). Waterpipe smoking: prevalence and attitudes among medical students in London [short communication]. Int J Tuberc Lung Dis.

[33] Akl E, et al. The allure of the waterpipe: a narrative review of factors affecting the epidemic rise in waterpipe smoking among young persons globally. Tob Control. 2015;24:i13-i21. 10.1136/tobaccocontrol-2014-051906 PMC4345979 25618895

[34] Jawad M, et al. Waterpipe tobacco smoking legislation and policy enactment: a global analysis. Tob Control. 2015;24:i60-i65. 10.1136/tobaccocontrol-2014-051911 PMC4345984 25550418

[35] Obeidat SR (2014). Prevalence, social acceptance, and awareness of waterpipe smoking among dental university students: a cross sectional survey conducted in Jordan. BMC Res Notes.

[36] Afifi R (2013). Social norms and attitudes linked to waterpipe use in the eastern Mediterranean region. Soc Sci Med.

[37] Nakkash RT, Khalil J, Afifi RA (2011). The rise in narghile (shisha, hookah) waterpipe tobacco smoking: a qualitative study of perceptions of smokers and non smokers. BMC Public Health.

[38] Salameh P (2014). Waterpipe dependence in university students and effect of normative beliefs: a cross-sectional study. BMC Open.

[39] Grekin ER, Ayna D (2012). Waterpipe smoking among college students in the United States: a review of the literature. J Am Coll Heal.

[40] Soule EK, Lipato T, Eissenberg T (2015). Waterpipe tobacco-smoking: a new smoking epidemic among the young?. Curr Pulmonol Rep.

[41] Maziak W (2014). The waterpipe: a new way of hooking youth on tobacco. Am J Addict.

[42] Akl EA (2013). Motives, beliefs and attitudes towards waterpipe tobacco smoking: a systematic review. Harm Reduct J.

[43] Musmar S (2012). Smoking habits and attitudes among university students in Palestine: a cross-sectional study. East Mediterr Health J.

[44] Wetter DW (2004). Prevalence and predictors of transitions in smoking behavior among college students. Health Psychol.

[45] Roohafza H (2015). Smoking motivators are different among cigarette and waterpipe smokers: the results of ITUPP. J Epidemiol Glob Health.

[46] Shomar RTA (2014). Smoking, awareness of smoking-associated health risks, and knowledge of national tobacco legislation in Gaza*,* Palestine. Cent Eur J Public Health.

[47] Khalil J (2013). Women and waterpipe tobacco smoking in the eastern Mediterranean region: allure or offensiveness. Women Health.

[48] Hammal F, et al. A pleasure among friends: how narghile (waterpipe) smoking differs from cigarette smoking in Syria. Tob Control. 2008;17(2):p. e3-e3. 10.1136/tc.2007.020529 18375726

[49] Maziak W, Jawad M, Jawad S, Ward KD, Eissenberg T, Asfar T. Interventions for waterpipe smoking cessation. Cochrane Database of Syst Rev. 2015;(7). Art.No.:CD005549. doi:10.1002/14651858.CD005549.pub3.51. 10.1002/14651858.CD005549.pub3 PMC4838024 26228266

[50] Eissenberg T (2008). Waterpipe tobacco smoking on a US College campus: prevalence and correlates. J Adolescent Health.

[51] Xuan LTT, Van Minh H, Giang KB, Nga PTQ, Hai PT, Minh NT, et al. Prevalence of Waterpipe Tobacco Smoking Among Population Aged 15 Years or Older, Vietnam, 2010. Prev Chronic Dis. 2013;10:120100. http://dx.doi.org/10.5888/pcd10.120100. 10.5888/pcd10.120100 PMC3638612 23597395

[52] Wechsler H (1998). Increased levels of cigarette use among college students: a cause for national concern. JAMA.

[53] Primack BA (2013). Waterpipe smoking among US university students. Nicotine Tob Res.

[54] Husseini, A. Smoking and associated factors in the occupied Palestinian territory. Palestine: Institute of Community and Public Health, Birzeit University; 2010.

[55] Roberts B (2013). Tobacco use and nicotine dependence among conflict-affected men in the republic of Georgia. Int J Environ Res Public Health.

[56] Abu-Rmeileh NM, et al. Smoking among adolescents and teenagers living under conflict: cross-sectional surveys in three settings. The Lancet [Abstract], 2011.

[57] Roberts ME (2008). Association between trauma exposure and smoking in a population-based sample of young adults. J Adolesc Health.

[58] Bailey S, Murray D. The status of youth in Palestine. 2009. Sharek Youth Forum.

[59] Alzyoud S (2013). Waterpipe smoking among middle and high school Jordanian students: patterns and predictors. Int J Environ Res Public Health.

[60] Pennanen M (2011). Longitudinal study of relations between school achievement and smoking behavior among secondary school students in Finland: results of the ESFA study. Subst Use Misuse.

[61] Ministry of Health, Palestinian National Health Strategy 2011–2013: Setting Direction - Getting Results 2010.

[62] Palestinian Legislative Council Palestinian Legislative Council - Public Health Law. 2005.

