# Supplementary material for: Waterpipe and cigarette tobacco smoking among Palestinian university students: a cross-sectional study
Source: BMC Public Health. 2017 Jul 10;18:1. doi: 10.1186/s12889-017-4524-0 (PMC5504745; doi:10.1186/s12889-017-4524-0)
Supplement: Additional file 1: — Logistic regression for current waterpipe tobacco smoking and cigarette smoking by participants’ characteristics for men in the sample only (n = 929) and women in the sample only (n = 962). (PDF 146 kb) [file 12889_2017_4524_MOESM1_ESM.pdf]

Table 4 Logistic regression for current waterpipe tobacco smoking and cigarette smoking by participants' characteristics for men in the sample only (n = 929) and women in the sample only (n = 962)

Characteristic	Waterpipe tobacco smoking		Cigarette smoking	
	Men	Women	Men	Women
	AOR (95% CI)	AOR (95% CI)	AOR (95% CI)	AOR (95% CI)
Locality				
Urban & Camp [R]	-	-	1	-
Rural	-	-	2.08 (1.24–3.51)	-
Geographic area of residence				
Gaza Strip [R]	1	1	1	1
North West Bank	2.58 (1.80–3.68)	7.23 (3.16–16.53)	4.48 (2.96–6.79)	5.65 (1.32–24.13)
Central West Bank	1.53 (1.02–2.31)	9.54 (4.64–19.65)	2.45 (1.54–3.88)	8.71 (2.47–30.79)
South West Bank	2.31 (1.45–3.66)	6.43 (3.17–13.02)	3.00 (1.79–5.05)	4.41 (1.20–16.24)
Living arrangement				
With family [R]	-	-	-	-
Other	-	-	-	-
Employment status				
Not employed [R]	-	-	-	-
Employed	-	-	-	-
Father's highest educational attainment				
Less than high school [R]	-	-	-	1
High school & above	-	-	-	4.78 (1.12–20.29)
Mother's highest educational attainment				
Less than high school [R]	-	-	-	-
High school & above	-	-	-	-
Self-reported economic standing				
Poor [R]	1	-	-	-
Good	1.32 (0.93–1.88)	-	-	-
Very good	1.84 (1.27–2.66)	-	-	-
Current faculty of study				
Sciences & health sciences [R]	-	-	1	-
Arts & humanities	-	-	1.44 (1.04–1.99)	-
Self-reported academic achievement				
GPA[b] ≥ 80.0 [R]	1	1	1	-
GPA 70.0–79.9	1.92 (1.36–2.72)	1.76 (1.12–2.75)	2.39 (1.61–3.54)	-
GPA ≤ 69.9	2.89 (1.79–4.67)	3.76 (1.82–7.63)	5.87 (3.46–9.99)	-

[R]Reference category
[b]GPA Grade point average
